# Associations of a body shape index, weight-adjusted waist index with the activities of daily living impairment in the middle-aged and elderly population: results from the China Health and Retirement Longitudinal Study

**DOI:** 10.3389/fmed.2025.1519362

**Published:** 2025-05-07

**Authors:** Yihang Du, Wenjing Zhang, Zizhen Chen, Xueping Zhu, Meng Lyu, Yi Wei, Yuanhui Hu

**Affiliations:** Department of Cardiology, Guang’anmen Hospital, China Academy of Chinese Medical Sciences, Beijing, China

**Keywords:** a body shape index, weight-adjusted waist index, activities of daily living, central obesity, China Health and Retirement Longitudinal Study

## Abstract

**Objective:**

To investigate and compare the associations of body mass index (BMI), waist circumference (WC), a body shape index (ABSI), weight-adjusted waist index (WWI), and waist-to-height ratio (WHtR) with activities of daily living (ADL) impairment among the middle-aged and elderly population in China.

**Methods:**

In this study, 8,700 participants from 2011 were included in the cross-section analysis. The prospective study used baseline data from 2011 and follow-up data from 2013, 2015, 2018, and 2020, with a total of 5,945 participants included. Binary logistic regression models were employed in the cross-sectional study to calculate odds ratio (OR) with corresponding 95% confidence interval (95% CI). In the prospective study, Cox proportional hazards regression models were utilized to estimate hazard ratio (HR) with 95% CI. Restricted cubic spline curve was used to identify trends, with subgroup analysis performed. Diagnostic performance was quantified through receiver operating characteristic curve analysis, with area under the curve values computed.

**Results:**

In the cross-sectional study, BMI (OR = 0.98, 95% CI: 0.97–1.00) was significantly negatively associated with ADL impairment risk, while ABSI (OR = 1.25, 95% CI: 1.14–1.37), WWI (OR = 1.16, 95% CI: 1.09–1.24) were positively associated with ADL impairment. However, there was no significant association between WC, WHtR and ADL impairment. In the prospective study, WC, WHtR and BMI showed no significant association with ADL impairment, while ABSI (HR = 1.07, 95% CI: 1.01–1.14) and WWI (HR = 1.05, 95% CI: 1.01–1.10) remained positively associated with ADL impairment. Positive linear correlations were observed among ABSI, WWI, and ADL impairment and there was no difference among subgroup variables. The predictive ability of WWI was slightly higher than ABSI, with an AUC value of 0.598.

**Conclusion:**

ABSI and WWI were significantly associated with the risk of ADL impairment among the middle-aged and elderly population in China. ABSI and WWI had the potential to serve as superior obesity indicators for identifying individuals at higher risk for ADL impairment.

## 1 Introduction

Activities of daily living (ADL) refer to the essential activities necessary for an individual to maintain independent life, such as dressing, eating, bathing, toileting, mobility, and community activities. Assessment of ADL is an important indicator of self-care ability, health status, and quality of life for middle-aged and elderly people (1). The decline in ADL may lead to an increased risk of falls (2), worsening of geriatric syndromes (such as frailty, cognitive decline, depression, etc.) (3), and is associated with higher hospitalization rates and mortality (4). It is projected that by 2060, the number of middle-aged and elderly individuals with ADL limitations or functional decline will increase to 96.2 million, imposing a huge economic burden on individuals, health systems and care resources (5, 6). Therefore, it is essential to regularly assess ADL function and identify potential risk factors that lead to ADL impairment.

Obesity significantly impairs ADL and reduces the ability to live independently and overall quality of life through multiple pathways, such as weakening muscle strength, reducing physical flexibility, and promoting chronic diseases (7). Body Mass Index (BMI) is a commonly used indicator for assessing the degree of obesity. However, BMI ignores the differences in the ratio of muscle to fat and the distribution of fat, which leads to incorrect assessments of muscular individuals (8). Compared to BMI, the assessment of central obesity can more accurately reflect the distribution of body fat, especially the accumulation of abdominal fat, which is more effective in predicting the risk of chronic diseases. Moreover, it is less affected by muscle mass factors and more accurately reflects the individual’s obesity status and health risks (9). Thus, central obesity is prone to trigger chronic diseases and functional decline, which in turn can lead to ADL impairment.

Waist circumference (WC) has been regarded as a more reliable indicator for assessing the risk of central obesity-related diseases. WC can more accurately reflect abdominal fat distribution and visceral obesity. However, WC does not take into account height variations, which may lead to biases in risk assessment for individuals of different heights (10). Therefore, it is not reliable to evaluate obesity risk only by BMI or WC. In recent years, novel anthropometric indices such as the a body shape index (ABSI) (11), weight-adjusted waist index (WWI) (12), and waist-to-height ratio (WHtR) (13) have emerged as valuable indicators in central obesity-related diseases. These comprehensive indicators exhibit superior predictive efficacy compared to conventional parameters, particularly in assessing central obesity-related health status and forecasting disease risks (14). Previous studies were mostly limited to cross-sectional research, and no prospective study has explored the association between central obesity and ADL impairment. Therefore, based on the China Health and Retirement Longitudinal Study (CHARLS), this study aimed to investigate and compare the associations between BMI, WC, ABSI, WWI, WHtR, and ADL impairment, further promote the application of the composite indicators of central obesity in the daily health monitoring of the middle - aged and elderly population.

## 2 Methods

### 2.1 Study participants

This study utilized data of CHARLS from 2011 to 2020 to explore the associations between BMI, WC, ABSI, WWI, WHtR, and ADL impairment in middle-aged and older adults aged 45 years and above. The study participants were representative samples from 28 provinces across China, selected using a stratified cluster sampling method (15). Baseline data covered information such as age, gender, height, weight, WC, education level, marital status, residence place, smoking, drinking, hypertension, diabetes mellitus (DM) and dyslipidemia. To ensure data completeness and accuracy, samples with missing data, outliers, or logical errors were excluded. Participants were excluded if they met the following criteria: (1) age below 45; (2) missing ADL data; (3) missing BMI, WC, ABSI, WWI, WHtR data; (4) missing demographic data; (5) WC less than 40 cm. Ultimately, 8,700 subjects from 2011 were included in the cross-section analysis. The prospective study used baseline data from 2011 and follow-up data from 2013, 2015, 2018, and 2020, with a total of 5,945 participants included. This study was approved by the Peking University Medical Ethics Committee (IRB00001052-11015), and all participants signed informed consent to participate in the follow-up surveys.

### 2.2 ADL impairment assessment

ADL impairment was mainly assessed using the ADL scale. Each item on the scale had four response options: “no difficulty, ““some difficulty but can still complete, ““difficulty and needs help, “and “unable to complete” (16). The scale included six tasks essential for independent living: eating, toileting, bathing, dressing, indoor activities, and continence control, as well as tasks related to community living, including housekeeping, cooking, shopping, medication use, telephone use, and managing finances. ADL impairment was defined as the inability to perform any of the above tasks independently (17).

### 2.3 Measurement of BMI, WC, ABSI, WWI, and WHtR

In the CHARLS study, measurements of WC, height, and weight were conducted following strict protocols. WC was measured using a calibrated tape measure at the midpoint between the lower edge of the rib cage and the iliac crest, with the subject standing in a natural breathing position and the reading taken at the end of expiration. Height was measured with the participant standing barefoot, with eyes looking straight ahead. For weight measurement, participants were required to remove heavy outer clothing and any accessories that might affect the results, and the value was recorded once stabilized. BMI was calculated using the formula: $BMI=\frac{W\mathrm{eight}}{\mathrm{Heigh}t^{2}}$ kg/m^2^, which is used to assess weight status (18). ABSI was calculated using the formula: $\mathrm{ABSI}=\frac{WC}{BMI^{2/3}\times \mathrm{Heigh}t^{1/2}}$ (19). WHtR was calculated by dividing WC by height, incorporating body shape into the assessment of central obesity. Finally, the WWI was derived by dividing WC by the square root of weight, reflecting the presence of excessive body fat buildup and the loss of muscular mass (14).

### 2.4 Covariates

To assess the impact of potential risk factors, demographic and health status variables were collected through a structured questionnaire. These included age (as a continuous variable), gender (male or female), marital status (married or non married), place of residence (urban or rural), education level (illiterate, primary/middle school, high school or above), smoking, and drinking (categorized as no or yes based on current behavior).

### 2.5 Health status assessment

Hypertension diagnosis was based on blood pressure measurements and physician diagnosis. Dyslipidemia was diagnosed if any of the following criteria were met: total cholesterol ≥6.2 mmol/L, low-density lipoprotein cholesterol ≥4.1 mmol/L, triglycerides ≥2.3 mmol/L, High-density lipoprotein cholesterol <1 mmol/L, or physician diagnosis (20). The diagnosis criteria for DM included any of the following: fasting blood glucose ≥7 mmol/L, random blood glucose ≥11.1 mmol/L, glycated hemoglobin ≥6%, or physician diagnosis (21).

### 2.6 Statistical strategy

Participants were divided into two groups based on the presence or absence of ADL impairment. Means±standard deviations were used to represent continuous variables, while percentages were used for categorical variables. T-test was used for continuous variables, and a chi-square test was used to assess differences between groups for categorical variables. Binary logistic regression models were employed in the cross-sectional study to calculate odds ratio (OR) with corresponding 95% confidence interval (95% CI). In the prospective study, Cox proportional hazards regression models were utilized to estimate hazard ratio (HR) with 95% CI, complemented by Kaplan–Meier (KM) survival curve visualization. To control for potential confounding factors, adjustments were made: Model 1 was adjusted for age and gender; Model 2 was further adjusted for smoking, alcohol consumption, residence, marital status, and education level; Model 3 was additionally adjusted for hypertension, dyslipidemia, and DM. Subsequently, restricted cubic spline (RCS) curve was used to identify trends, with subgroup analyses performed according to gender, marital status, residence, smoking, drinking, hypertension, dyslipidemia, DM, BMI and age. For enhanced clinical interpretability of OR and HR, ABSI values were standardized by multiplying by 100. Similarly, WHtR values were standardized by multiplying by 10. Diagnostic performance was quantified through receiver operating characteristic (ROC) curve analysis, with area under the curve (AUC) values computed. The statistical significance set at *p* < 0.05. All statistical analyses were performed using R 4.4.1.

## 3 Results

### 3.1 Baseline characteristics of the participants in the cross-sectional study

Table 1 presents the baseline characteristics of the 8,700 participants. Among all participants, 3,603 (41.41%) were male, and 5,097 (58.59%) were female. The results indicated that 1,776 participants suffered from ADL impairment. Compared to those without ADL impairment, participants with ADL impairment were generally older, female, unmarried, living in rural area, no drinking, less educated, and more likely to have hypertension, and DM, lower weight and BMI, while higher ABSI WHtR and WWI. However, no significant difference in WC was observed between the two groups.

**Table 1:** Baseline characteristics of participants in cross-sectional study.

Variable	Total (*n* = 8,700)	Non-impairment (*n* = 6,924)	Impairment (*n* = 1776)	Statistic	*p*
Age (year)	61.02 ± 9.83	60.14 ± 9.51	64.47 ± 10.28	−16.09	<0.0001
Sex				5.65	0.02
Female	5,097 (58.59)	4,012 (57.94)	1,085 (61.09)		
Male	3,603 (41.41)	2,912 (42.06)	691 (38.91)		
Marital status				34.65	<0.0001
Married	7,525 (86.49)	6,065 (87.59)	1,460 (82.21)		
Non married	1,175 (13.51)	859 (12.41)	316 (17.79)		
Education				200.24	<0.0001
High school or above	645 (7.41)	570 (8.23)	75 (4.22)		
Illiterate	4,655 (53.51)	3,441 (49.70)	1,214 (68.36)		
Primary/Middle school	3,400 (39.08)	2,913 (42.07)	487 (27.42)		
Residence place				16.41	<0.0001
Rural	5,743 (66.01)	4,498 (64.96)	1,245 (70.10)		
Urban	2,957 (33.99)	2,426 (35.04)	531 (29.90)		
Smoke				0.83	0.36
No	6,276 (72.14)	4,979 (71.91)	1,297 (73.03)		
Yes	2,424 (27.86)	1945 (28.09)	479 (26.97)		
Drink				21.66	<0.0001
No	6,260 (71.95)	4,903 (70.81)	1,357 (76.41)		
Yes	2,440 (28.05)	2021 (29.19)	419 (23.59)		
Hypertension				31.07	<0.0001
No	4,838 (55.61)	3,955 (57.12)	883 (49.72)		
Yes	3,862 (44.39)	2,969 (42.88)	893 (50.28)		
Dyslipidemia				3.40	0.07
No	5,825 (66.95)	4,669 (67.43)	1,156 (65.09)		
Yes	2,875 (33.05)	2,255 (32.57)	620 (34.91)		
DM				9.17	<0.01
No	7,485 (86.03)	5,997 (86.61)	1,488 (83.78)		
Yes	1,215 (13.97)	927 (13.39)	288 (16.22)		
WC (cm)	85.69 ± 10.52	85.69 ± 10.35	85.72 ± 11.14	−0.10	0.92
Weight (kg)	58.03 ± 11.84	58.56 ± 11.80	55.97 ± 11.78	8.28	<0.0001
Height (m)	1.57 ± 0.09	1.57 ± 0.08	1.55 ± 0.09	7.80	<0.0001
BMI (kg/m^2^)	23.50 ± 4.05	23.61 ± 4.07	23.07 ± 3.95	5.12	<0.0001
ABSI	0.08 ± 0.01	0.08 ± 0.01	0.09 ± 0.01	−10.24	<0.0001
WWI	11.32 ± 0.94	11.26 ± 0.91	11.53 ± 1.03	−10.08	<0.0001
WHtR	0.55 ± 0.07	0.55 ± 0.07	0.55 ± 0.07	−3.54	<0.001

DM, diabetes mellitus; WC: waist circumference; BMI, body mass index; ABSI, a body shape index; WWI, weight-adjusted waist index; WHtR, waist-to-height ratio.

### 3.2 Associations of BMI, ABSI, WWI, and WHtR with ADL impairment in the cross-sectional study

Table 2 shows the associations between BMI, ABSI, WWI, WHtR, and ADL impairment using the binary logistic regression model. After adjusting for age, gender, smoking, drinking, residence place, marital status, education, hypertension, dyslipidemia, and DM, BMI (OR = 0.98, 95% CI: 0.97–1.00) showed negative association with ADL impairment while ABSI (OR = 1.25, 95% CI: 1.14–1.37), WWI (OR = 1.16, 95% CI: 1.09–1.24) were positively associated with ADL impairment.

**Table 2:** Binary logistic regression analysis of BMI, ABSI, WWI and WHtR with ADL impairment in cross-sectional study.

Character	Crude model		Model 1		Model 2		Model 3	
	OR (95% CI)	*p*	OR (95% CI)	*p*	95% CI	*p*	OR (95% CI)	*p*
BMI	0.97 (0.95, 0.98)	<0.0001	0.98 (0.97, 1.00)	0.03	0.99 (0.98, 1.01)	0.35	0.98 (0.97, 1.00)	0.03
Quartile 1	Reference		Reference		Reference		Reference	
Quartile 2	0.82 (0.71, 0.94)	0.01	0.92 (0.79, 1.06)	0.26	0.96 (0.83, 1.11)	0.58	0.93 (0.80, 1.08)	0.33
Quartile 3	0.71 (0.61, 0.82)	<0.0001	0.83 (0.71, 0.96)	0.01	0.9 (0.77, 1.04)	0.16	0.84 (0.72, 0.99)	0.03
Quartile 4	0.72 (0.62, 0.83)	<0.0001	0.88 (0.75, 1.02)	0.09	0.96 (0.82, 1.13)	0.64	0.86 (0.73, 1.02)	0.08
*p* for trend		<0.0001		0.04		0.46		0.04
ABSI	1.59 (1.46, 1.73)	<0.0001	1.25 (1.14, 1.37)	<0.0001	1.26 (1.15, 1.38)	<0.0001	1.25 (1.14, 1.37)	<0.0001
Quartile 1	Reference		Reference		Reference		Reference	
Quartile 2	1.2 (1.01, 1.42)	0.03	1.13 (0.95, 1.33)	0.16	1.15 (0.97, 1.36)	0.11	1.14 (0.96, 1.35)	0.13
Quartile 3	1.47 (1.26, 1.72)	<0.0001	1.24 (1.06, 1.45)	0.01	1.27 (1.08, 1.48)	0.004	1.24 (1.06, 1.45)	0.01
Quartile 4	2.09 (1.79, 2.43)	<0.0001	1.41 (1.20, 1.66)	<0.0001	1.45 (1.23, 1.71)	<0.0001	1.42 (1.20, 1.67)	<0.0001
*p* for trend		<0.0001		<0.0001		<0.0001		<0.0001
WWI	1.35 (1.28, 1.43)	<0.0001	1.17 (1.10, 1.25)	<0.0001	1.18 (1.11, 1.26)	<0.0001	1.16 (1.09, 1.24)	<0.0001
Quartile 1	Reference		Reference		Reference		Reference	
Quartile 2	0.97 (0.83, 1.14)	0.74	0.92 (0.78, 1.08)	0.33	0.94 (0.80, 1.11)	0.49	0.92 (0.78, 1.09)	0.33
Quartile 3	1.32 (1.13, 1.54)	<0.001	1.15 (0.98, 1.35)	0.08	1.17 (0.99, 1.37)	0.06	1.12 (0.96, 1.32)	0.16
Quartile 4	1.89 (1.63, 2.19)	<0.0001	1.35 (1.14, 1.59)	<0.001	1.38 (1.17, 1.63)	<0.001	1.32 (1.11, 1.56)	0.002
*p* for trend		<0.0001		<0.0001		<0.0001		<0.001
WHtR	1.15 (1.07, 1.24)	<0.001	1.07 (0.99, 1.16)	0.09	1.12 (1.03, 1.22)	0.01	1.08 (0.99, 1.17)	0.09
Quartile 1	Reference		Reference		Reference		Reference	
Quartile 2	0.97 (0.84, 1.13)	0.72	0.96 (0.83, 1.12)	0.63	1 (0.86, 1.17)	0.97	0.98 (0.84, 1.15)	0.80
Quartile 3	1.08 (0.93, 1.26)	0.30	1.05 (0.90, 1.22)	0.56	1.12 (0.95, 1.31)	0.17	1.06 (0.91, 1.25)	0.45
Quartile 4	1.24 (1.07, 1.44)	0.004	1.1 (0.94, 1.29)	0.23	1.2 (1.02, 1.41)	0.03	1.11 (0.93, 1.31)	0.24
*p* for trend		0.001		0.14		0.01		0.16

Model 1, age, and sex were adjusted; Model 2, age, sex, smoke, drink, residence place, marital status, education were adjusted; Model 3, age, sex, smoke, drink, residence place, marital status, education, hypertension, dyslipidemia, DM were adjusted.

### 3.3 Baseline characteristics of the participants in the prospective study

The baseline population in 2011 consisted of 5,945 participants. 3,096 participants had experienced ADL impairment during the follow-up period. Among them, 2,392 participants (40.24%) were male, and 3,553 participants (59.76%) were female. Among all participants, 3,202 experienced a complete 9-year follow-up period. The results indicated that participants with ADL impairment tended to be older, female, unmarried, less educated, living in rural areas, non-smoker, non-drinker, and were more likely to have hypertension and DM. Additionally, these participants had lower BMI and higher ABSI, WHtR, WWI compared to those without ADL impairment. However, there was no significant difference of WC between the two groups (Table 3).

**Table 3:** Baseline characteristics of participants in the prospective study.

Variable	Total (*n* = 5,945)	Non-impairment (*n* = 2,849)	impairment (*n* = 3,096)	Statistic	*p*
Age (year)	59.66 ± 9.26	56.26 ± 7.58	62.79 ± 9.56	−29.31	<0.0001
Sex				48.24	<0.0001
Female	3,553 (59.76)	1,571 (55.14)	1982 (64.02)		
Male	2,392 (40.24)	1,278 (44.86)	1,114 (35.98)		
Marital status				136.86	<0.0001
Married	5,252 (88.34)	2,662 (93.44)	2,590 (83.66)		
Non married	693 (11.66)	187 (6.56)	506 (16.34)		
Education				430.62	<0.0001
High school or above	448 (7.54)	328 (11.51)	120 (3.88)		
Illiterate	3,017 (50.75)	1,061 (37.24)	1956 (63.18)		
Primary/Middle school	2,480 (41.72)	1,460 (51.25)	1,020 (32.95)		
Residence place				39.95	<0.0001
Rural	4,032 (67.82)	1818 (63.81)	2,214 (71.51)		
Urban	1913 (32.18)	1,031 (36.19)	882 (28.49)		
Smoke				7.21	<0.01
No	4,335 (72.92)	2031 (71.29)	2,304 (74.42)		
Yes	1,610 (27.08)	818 (28.71)	792 (25.58)		
Drink				29.06	<0.0001
No	4,241 (71.34)	1938 (68.02)	2,303 (74.39)		
Yes	1704 (28.66)	911 (31.98)	793 (25.61)		
Hypertension				75.67	<0.0001
No	3,473 (58.42)	1830 (64.23)	1,643 (53.07)		
Yes	2,472 (41.58)	1,019 (35.77)	1,453 (46.93)		
Dyslipidemia				0.76	0.38
No	3,987 (67.06)	1927 (67.64)	2060 (66.54)		
Yes	1958 (32.94)	922 (32.36)	1,036 (33.46)		
DM				7.05	<0.01
No	5,171 (86.98)	2,513 (88.21)	2,658 (85.85)		
Yes	774 (13.02)	336 (11.79)	438 (14.15)		
WC (cm)	85.68 ± 10.29	85.90 ± 9.99	85.47 ± 10.56	1.63	0.10
Weight (kg)	58.53 ± 11.71	60.43 ± 11.34	56.79 ± 11.78	12.14	<0.0001
Height (m)	1.57 ± 0.08	1.59 ± 0.08	1.56 ± 0.08	13.98	<0.0001
BMI (kg/m^2^)	23.66 ± 4.06	23.97 ± 3.88	23.38 ± 4.20	5.66	<0.0001
ABSI	0.08 ± 0.01	0.08 ± 0.01	0.08 ± 0.01	−12.34	<0.0001
WWI	11.26 ± 0.90	11.41 ± 0.94	11.10 ± 0.83	−13.55	<0.0001
WHtR	0.55 ± 0.07	0.55 ± 0.07	0.54 ± 0.06	−4.45	<0.0001

DM, diabetes mellitus; WC: waist circumference; BMI, body mass index; ABSI, a body shape index; WWI, weight-adjusted waist index; WHtR, waist-to-height ratio.

### 3.4 Associations of BMI, ABSI, WWI, and WHtR with ADL impairment in the prospective study

The prospective associations between BMI, ABSI, WWI, WHtR and ADL impairment are presented in Table 4. After adjusting for confounding factors, both ABSI (HR = 1.07, 95% CI: 1.01–1.14) and WWI (HR = 1.05, 95% CI: 1.01–1.10) retained their significant associations with ADL impairment. The results of quartile analysis indicated that, in comparison with the lowest quartile, the highest quartile of WWI (HR = 1.14, 95% CI: 1.02–1.28) was significantly associated with an elevated risk of ADL impairment.

**Table 4:** Cox regression analysis of BMI, ABSI, WWI, and WHtR with ADL impairment in the prospective study.

Character	Crude model		Model 1		Model 2		Model 3	
	HR (95% CI)	*p*	HR (95% CI)	*p*	HR (95% CI)	*p*	HR (95% CI)	*p*
BMI	0.97 (0.96, 0.98)	<0.0001	0.99 (0.98, 1.00)	0.004	1 (0.99, 1.01)	0.43	0.99 (0.98, 1.00)	0.11
Quartile 1	Reference		Reference		Reference		Reference	
Quartile 2	0.79 (0.72, 0.87)	<0.0001	0.89 (0.81, 0.98)	0.02	0.93 (0.85, 1.03)	0.17	0.92 (0.83, 1.01)	0.09
Quartile 3	0.71 (0.64, 0.78)	<0.0001	0.82 (0.74, 0.90)	<0.0001	0.89 (0.81, 0.99)	0.03	0.87 (0.79, 0.97)	0.01
Quartile 4	0.72 (0.66, 0.80)	<0.0001	0.88 (0.79, 0.97)	0.01	0.97 (0.88, 1.08)	0.62	0.93 (0.83, 1.04)	0.19
*p* for trend		<0.0001		0.002		0.42		0.11
ABSI	1.48 (1.40, 1.57)	<0.0001	1.07 (1.01, 1.14)	0.02	1.08 (1.02, 1.14)	0.01	1.07 (1.01, 1.14)	0.02
Quartile 1	Reference		Reference		Reference		Reference	
Quartile 2	1.06 (0.95, 1.18)	0.27	0.98 (0.88, 1.09)	0.68	1 (0.90, 1.11)	0.96	0.99 (0.89, 1.11)	0.90
Quartile 3	1.24 (1.12, 1.37)	<0.0001	0.99 (0.90, 1.10)	0.87	1.01 (0.91, 1.12)	0.87	1 (0.90, 1.11)	1.00
Quartile 4	1.86 (1.68, 2.05)	<0.0001	1.1 (0.99, 1.22)	0.08	1.12 (1.00, 1.24)	0.04	1.11 (1.00, 1.23)	0.06
*p* for trend		<0.0001		0.09		0.05		0.07
WWI	1.33 (1.28, 1.38)	<0.0001	1.05 (1.01, 1.10)	0.02	1.06 (1.02, 1.11)	0.01	1.05 (1.01, 1.10)	0.02
Quartile 1	Reference		Reference		Reference		Reference	
Quartile 2	1.1 (0.99, 1.22)	0.08	1.01 (0.91, 1.12)	0.88	1.03 (0.92, 1.15)	0.61	1.02 (0.91, 1.13)	0.77
Quartile 3	1.31 (1.18, 1.45)	<0.0001	1.06 (0.95, 1.18)	0.30	1.08 (0.97, 1.20)	0.18	1.06 (0.95, 1.18)	0.28
Quartile 4	1.88 (1.70, 2.07)	<0.0001	1.14 (1.02, 1.28)	0.02	1.16 (1.04, 1.30)	0.01	1.14 (1.02, 1.28)	0.02
*p* for trend		<0.0001		0.01		0.005		0.01
WHtR	1.12 (1.07, 1.18)	<0.0001	0.98 (0.93, 1.04)	0.54	1.03 (0.98, 1.09)	0.27	1.01 (0.95, 1.07)	0.74
Quartile 1	Reference		Reference		Reference		Reference	
Quartile 2	1.07 (0.97, 1.18)	0.20	1.04 (0.94, 1.15)	0.45	1.07 (0.97, 1.19)	0.17	1.06 (0.96, 1.17)	0.26
Quartile 3	1.01 (0.91, 1.12)	0.89	0.94 (0.85, 1.04)	0.23	1 (0.90, 1.12)	0.94	0.98 (0.88, 1.09)	0.68
Quartile 4	1.21 (1.09, 1.33)	<0.001	0.98 (0.88, 1.08)	0.66	1.06 (0.96, 1.18)	0.25	1.02 (0.92, 1.14)	0.68
*p* for trend		0.002		0.31		0.48		0.96

Model 1, age, and sex were adjusted; Model 2, age, sex, smoke, drink, residence place, marital status, education were adjusted; Model 3, age, sex, smoke, drink, residence place, marital status, education, hypertension, dyslipidemia, DM were adjusted.

**Figure 1:**
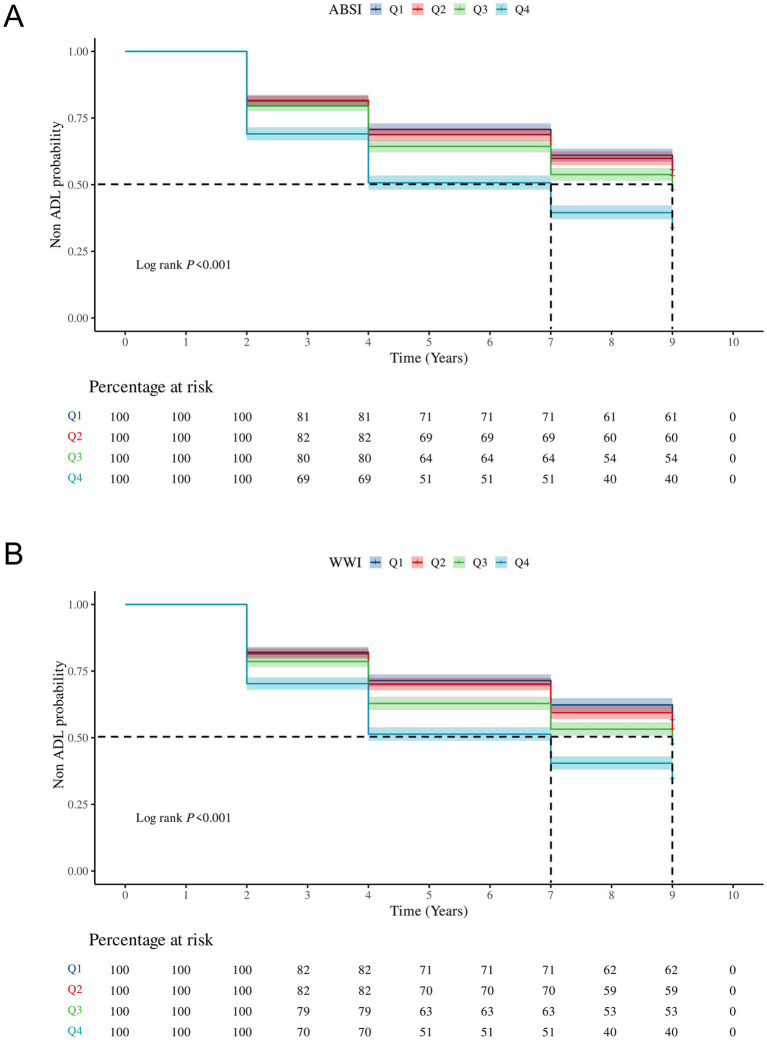
The KM survival curves for ADL impairment across quartile groups of ABSI **(A)** and WWI **(B)**. Q1, Quartile 1; Q2, Quartile 2; Q3, Quartile 3; Q4, Quartile 4; ADL, Activities of daily living; ABSI, A Body Shape Index; WWI, Weight-adjusted-waist index.

Figure 1 illustrates the KM survival curves for ADL impairment across quartile groups of ABSI and WWI. Notably, compared with the lowest quartile group, the risk of ADL impairment in the highest quartile group showed a more significant upward trend. Moreover, the median occurrence time for both ABSI and WWI in the highest quartile group was 7 years, and there was no significant difference between the two indicators.

### 3.5 RCS analysis in the prospective study

**Figure 2:**
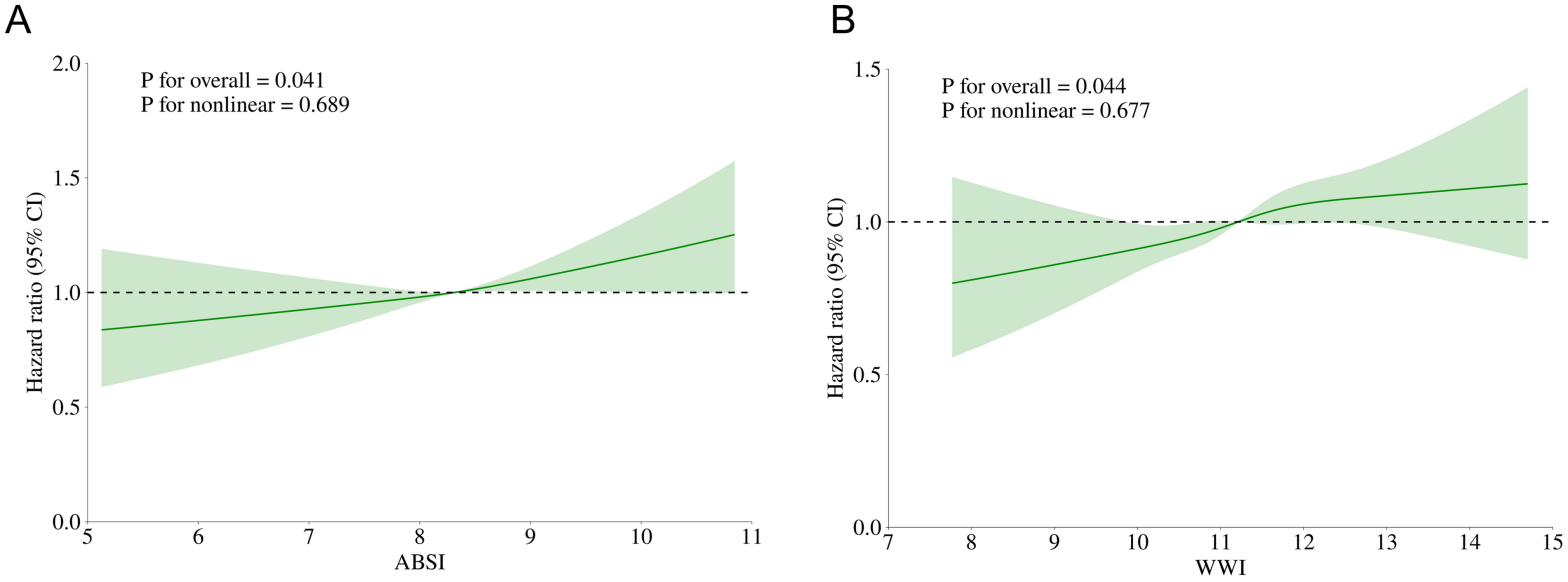
Restricted cubic splines of ABSI, WWI related to ADL impairment after adjusting for confounding factors. **(A)** RCS curve of ABSI related to ADL impairment; **(B)** RCS curve of WWI related to ADL impairment.

Figure 2 presents the results of the RCS analysis among ABSI, WWI, and ADL impairment in the prospective analysis. After adjustment for confounding factors, positive linear correlations were observed among ABSI, WWI, and ADL impairment, suggesting that as ABSI and WWI increase, the risk of ADL impairment occurring rises significantly.

### 3.6 Subgroup analysis in the prospective study

**Figure 3:**
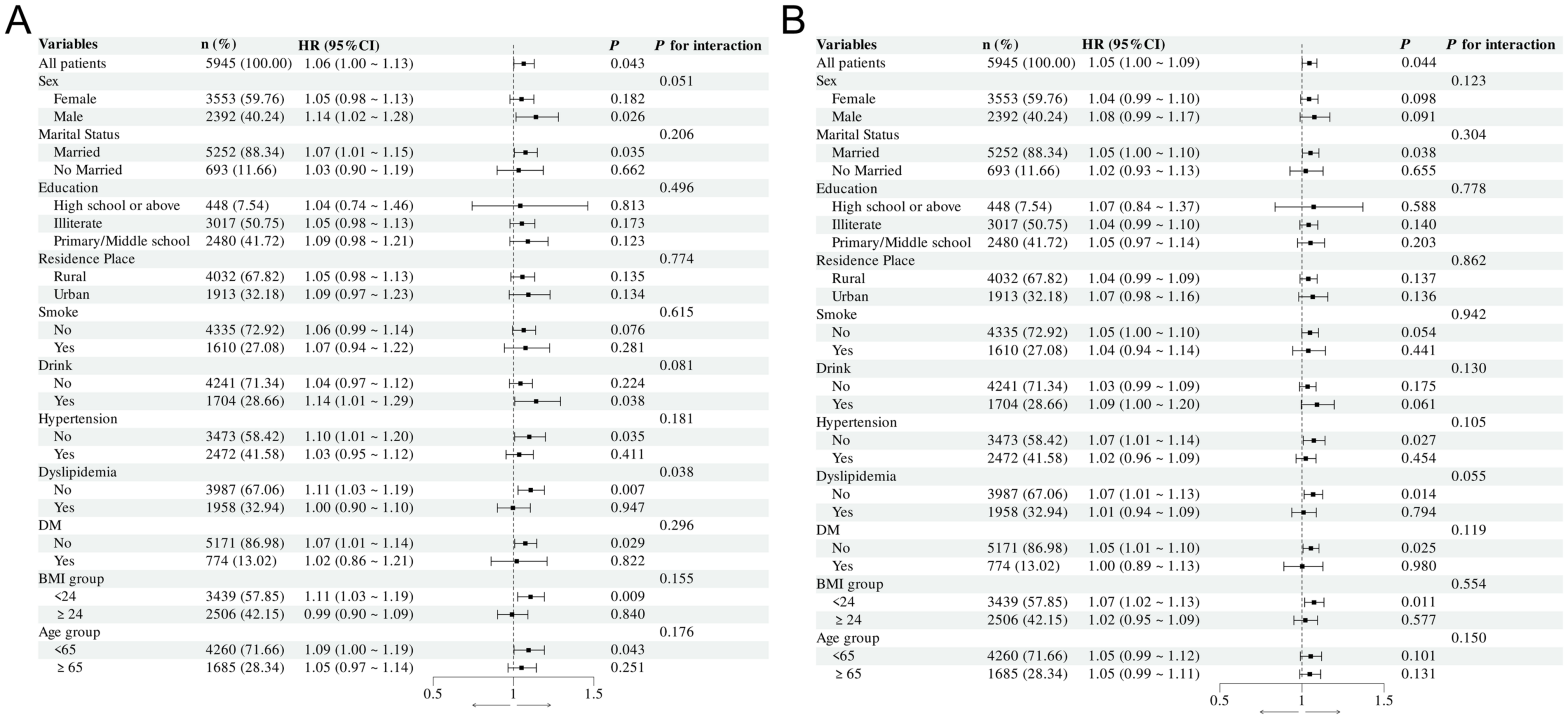
Forest plot of the relationship between ABSI, WWI and ADL impairment after adjusting for confounding factors. **(A)** Forest plot of the relationship between ABSI and ADL impairment in different subgroups; **(B)** Forest plot of the relationship between WWI and ADL impairment in different subgroups.

The results of the subgroup analysis indicates that there is no difference in the interaction among subgroup variables after adjusting for confounding factors, whether for ABSI or WWI. This implies that the impacts of ABSI and WWI on ADL impairment were generally stable (Figure 3).

### 3.7 ROC curve analysis in the prospective study

**Figure 4:**
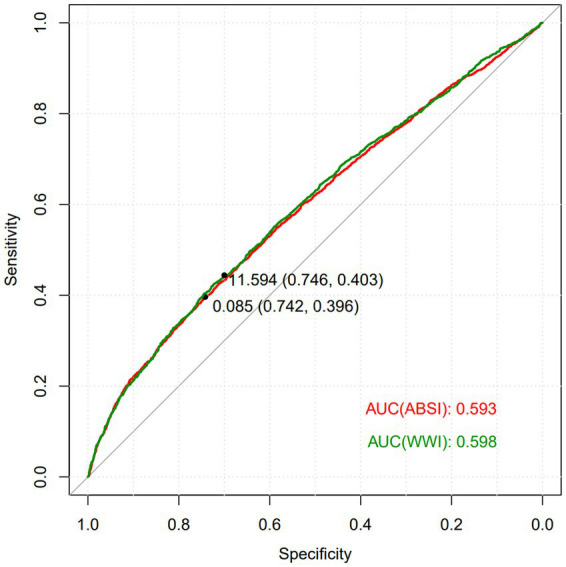
ROC curve of ABSI, WWI on ADL impairment.

The ROC curve was employed to evaluate the diagnostic value of ABSI and WWI in predicting ADL impairment. As depicted in Figure 4, the areas under the curves (AUC) of the two composite indices reflecting central obesity were both greater than 0.5. This indicates their potential in identifying the risk of ADL impairment. Notably, the predictive ability of WWI was slightly higher than ABSI, with an AUC value of 0.598.

## 4 Discussion

The results of this study indicated that as ABSI and WWI increases, the risk of ADL impairment rises significantly. ABSI and WWI were more effective indicators for predicting ADL impairment risk than BMI or WC.

ADL is typically used to evaluate an individual’s ability to independently perform basic daily tasks. Obesity increases the burden on joints, leading to arthritis and decreased mobility (22). Additionally, obesity is associated with decreased muscle mass, flexibility, and endurance (22, 23), which reduces mobility in elderly adults. It also weakens the individual’s functional independence by causing or aggravating a series of chronic diseases and psychological problems (24, 25). In fact, the use of BMI as a measure of obesity is limited because BMI neither determines the distribution of body fat nor distinguishes between muscle and adipose tissue (26). WC is commonly used to assess the degree of abdominal fat accumulation and central obesity, however, WC does not take into account height variations, which may lead to biases in risk assessment (10). WHtR is the ratio of WC to height, which not only considers the absolute value of waist circumference but also accounts for the influence of height, thereby reducing the biases in obesity assessment due to body shape (13). However, in this study, there was no significant association among BMI, WC, WHtR and ADL impairment. Thus, there is a need to introduce other indicators related to obesity to more sensitively capture health risks in individuals with different body types and ages.

ABSI, a novel body shape index proposed by Nir Krakauer (19), combines WC, height, and weight, with a special focus on abdominal fat distribution, which more accurately reflects central obesity and related health risks (19). ABSI has been found to be more effective than traditional BMI in predicting the risks of visceral fat-related cardiovascular diseases, hypertension, and DM (27–29). WWI is an indicator that integrates weight and WC information, and higher WWI may indicate a higher body fat percentage. WWI enhances the association with WC while weakening the association with weight, thus more comprehensively reflecting fat distribution and muscle mass (12). Changes in fat and muscle mass are closely related to the decline in physical function, with fat content being positively correlated with impairment (30). The decrease in muscle mass is often accompanied by an increase in body fat, especially among middle-aged and elderly population, and the combined changes of these two may more directly reflect the risk of functional impairment (31). A cross-sectional study from the Chinese Longitudinal Healthy Longevity Survey showed that among the elderly population (aged over 65 years), WWI was positively correlated with fat content and negatively correlated with weight, indicating that it is an excellent indicator reflecting the risk of obesity-related ADL impairment (31).

The ABSI and WWI were significantly associated with ADL impairment in this study, possibly because the ABSI and WWI can better track the dynamics of body fat distribution and differences across populations. In contrast, BMI is easily disturbed by short-term weight fluctuations and other factors, and cannot accurately reflect long-term trends. And WC only focuses on the localized situation of the abdomen, which may ignore the impact of the overall shape of the body and fat distribution patterns on ADL impairment (32).

However, this study has several limitations. Firstly, it may not have comprehensively controlled for all potential confounding factors that could influence the impairment of ADL, such as mental health status, social support, and nutritional intake. The incomplete control of these factors is likely to compromise the accuracy of the analysis results and introduce biases to the research conclusions. Secondly, during the research process, the dynamic changes of the ABSI and WWI over time were overlooked. There may be complex associations between ADL impairment and these two indices that change over time. Thirdly, this study collected data through self - reports using the ADL scale instead of objective indicators. The self - reporting method is vulnerable to the accuracy of recall.

Future research can further explore machine learning algorithms (such as random forest and gradient boosting tree) or hierarchical modeling strategies, and improve the prediction accuracy by optimizing feature selection and combination. Furthermore, this study has overlooked the distinct characteristics of the sarcopenic obesity population. In the future, in-depth research on the association between obesity and ADL impairment can be conducted in this specific population.

## 5 Conclusion

In summary, this study demonstrated that the risk of ADL impairment increased significantly with increasing ABSI and WWI among the middle-aged and elderly population in China, suggesting that ABSI and WWI may be valuable indicators for assessing the association between central obesity and ADL impairment.

## Data Availability

The original contributions presented in the study are included in the article/supplementary material, further inquiries can be directed to the corresponding authors.
